# Development and temporal validation of a machine learning-based prediction model for long-term depressive symptoms in older adults with cardiovascular disease or hypertension: A longitudinal cohort study

**DOI:** 10.1097/MD.0000000000050520

**Published:** 2026-09-18

**Authors:** Yang Zhao, Siji Chen, Chang Pang, Yao Tang, Si Long, Hua Zheng, Muhua Zeng, Shuwu Lin

**Affiliations:** a Shenyang Medical College, Shenyang, China; b Department of Heart Failure and Valvular Surgery, Beijing Anzhen Hospital, Capital Medical University, Beijing, China; c Jinzhou Medical University, Jinzhou, China; d First Department of Cadre Ward, The Second Affiliated Hospital of Shenyang Medical College, Shenyang, China.

**Keywords:** CHARLS, CVD, depressive symptoms, hypertension, machine learning model, older adults

## Abstract

Older adults with cardiovascular disease or hypertension have an elevated risk of depressive symptoms, which may adversely affect prognosis and quality of life. A practical prediction model could support early risk stratification and targeted screening. Data were obtained from the China Health and Retirement Longitudinal Study from 2011 to 2020. A time-based design was used for model development and temporal validation. The development cohort included 548 participants followed from 2011 to 2015, and the nonoverlapping temporal validation cohort included 523 participants followed from 2015 to 2020. The Boruta algorithm was used for predictor selection, 7 machine learning algorithms were compared, and Shapley additive explanations were used to interpret the selected model. Missing values were imputed using a random forest algorithm. Seven predictors were selected. Logistic regression showed the best overall performance, with an area under the receiver operating characteristic curve of 0.844 (95% confidence interval, 0.773–0.915) in the development cohort and 0.840 (95% confidence interval, 0.799–0.881) in the temporal validation cohort. The logistic regression model demonstrated good discrimination and calibration for predicting long-term depressive symptoms among older adults with cardiovascular disease or hypertension. Further independent and prospective validation is required before routine clinical implementation.

## 1. Introduction

Cardiovascular disease (CVD) encompasses a broad spectrum of conditions involving the heart and vascular system, such as coronary heart disease, heart failure, stroke, cardiomyopathy, arrhythmias, valvular disorders, peripheral arterial disease, and hypertensive heart disease.^[1]^ According to the World Health Organization, CVD accounts for more than 30% of global deaths,^[2]^ and this proportion is even higher in China.^[3]^ Furthermore, the WHO Global Report on Hypertension indicates that hypertension directly contributes to approximately 13% of deaths worldwide.^[4]^ With the accelerating aging of the population, an increasing number of older adults are living with CVD, hypertension, or both conditions, along with comorbid mental health issues. Depressive symptoms are not uncommon in older adults with CVD or hypertension, and their occurrence not only impairs quality of life but is also associated with higher rates of recurrent events, hospitalizations, and mortality.^[5]^ In the China Health and Retirement Longitudinal Study (CHARLS) cohort study, individuals with baseline depressive symptoms (assessed using the 10-item Center for Epidemiologic Studies Depression Scale [CES-D-10]) had a significantly increased risk of subsequent CVD (adjusted hazard ratio = 1.39; 95% confidence interval, 1.22–1.58). This evidence highlights the close relationship between psychological status and cardiovascular health.^[6]^ However, most existing studies have focused on the effect of depression on CVD, while prospective studies specifically examining the risk of developing depressive symptoms in older adults with CVD or hypertension remain relatively limited. From a pathophysiological perspective, multiple interconnected pathways may exist between CVD or hypertension and depressive symptoms. Older adults with CVD or hypertension often experience impaired daily functioning, restricted social participation, elevated chronic inflammation, reduced heart rate variability, and autonomic dysfunction. These physiological and psychosocial factors may collectively contribute to the onset and worsening of depressive symptoms.^[5,7]^ Conversely, depressive symptoms may promote cardiovascular progression through unhealthy lifestyle behaviors (such as smoking, sedentary behavior, and poor medication adherence) and biological mechanisms (such as high inflammation and endothelial dysfunction).^[8]^ Therefore, using the CHARLS database, this study developed a risk prediction model for depressive symptoms in older adults with CVD or hypertension by applying machine learning algorithms. The aim is to provide a basis for early identification of high-risk individuals and to offer insights for the management of cardiovascular psychiatric comorbidity.

## 2. Materials and methods

### 2.1. Data source

CHARLS is a nationally representative longitudinal survey of Chinese adults aged 45 years and older and their spouses. The study was conducted in accordance with the Declaration of Helsinki. To date, CHARLS has released 5 waves of data: the 2011 national baseline survey (Wave 1), the first follow-up in 2013 (Wave 2), the second follow-up in 2015 (Wave 3), the third follow-up in 2018 (Wave 4), and the fourth follow-up in 2020 (Wave 5). All waves received approval from the Institutional Review Board at Peking University, and all participants gave written informed consent. The ethics approval number for the main household survey (including anthropometric measurements) is IRB00001052-11015, and for biomarker collection is IRB00001052-11014.

### 2.2. Study population and cohort construction

**Figure 1.**
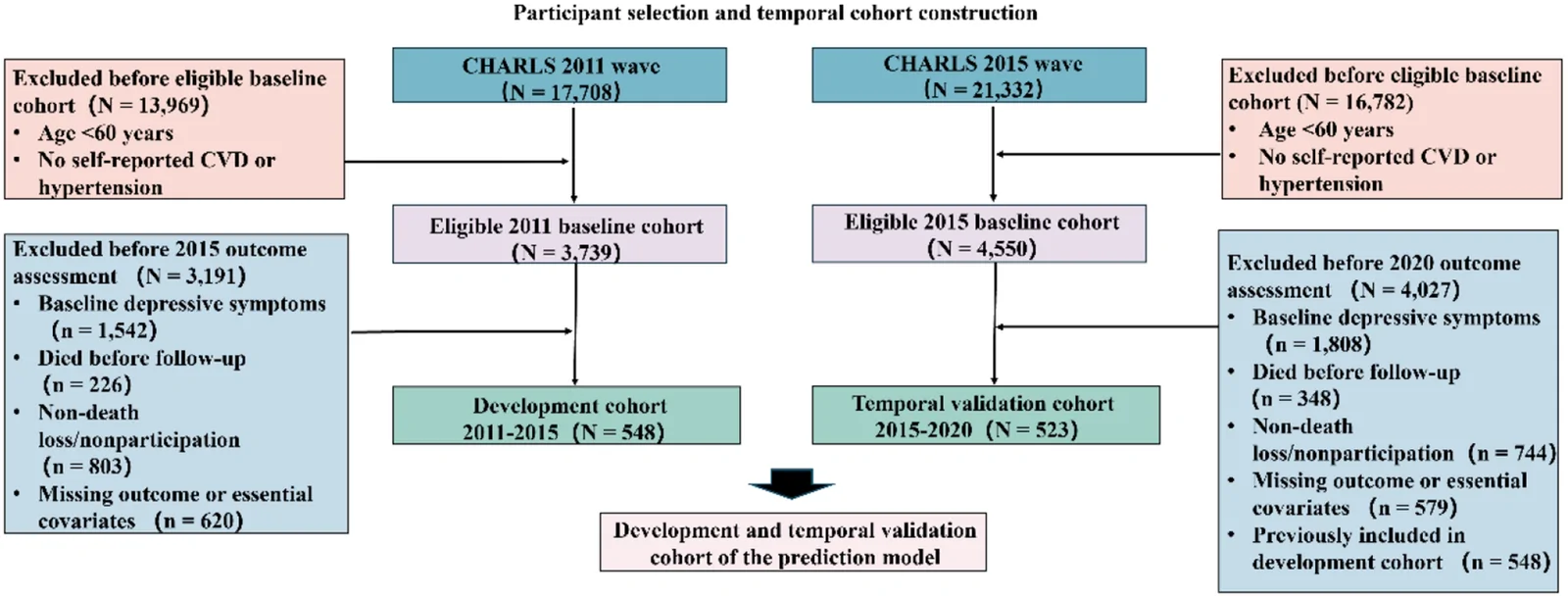
Flowchart of the study sample selection. CHARLS = China Health and Retirement Longitudinal Study, CVD = cardiovascular disease.

Participants were eligible if they: were aged ≥60 years at baseline; had CVD, hypertension, or both at baseline; had no depressive symptoms at baseline (CES-D score <10); completed the corresponding follow-up survey with available CES-D data; and had complete information on key predictor variables. The identification of CVD and hypertension was based on the standardized self-reported questionnaire data of individuals in CHARLS. CVD was defined as a self-reported physician diagnosis of heart disease or stroke, and hypertension was defined as a self-reported physician diagnosis of hypertension or ongoing use of antihypertensive medication. Blood pressure measurements obtained at the time of CHARLS were not employed to identify eligible participants. Participants were excluded if they: were younger than 60 years at baseline or did not meet the above definition of CVD or hypertension; had depressive symptoms at baseline (CES-D score ≥10); were lost to follow-up, died during follow-up, or had missing outcome data; or had missing values for essential covariates. To develop and validate the prediction model, a time-based cohort division strategy was used. Participants from the 2011 CHARLS survey who were free of depressive symptoms at baseline and completed the 2015 follow-up assessment were assigned to the development cohort. Participants from the 2015 survey who met the same eligibility criteria and completed the 2020 follow-up assessment were assigned to the temporal validation cohort. Individuals included in the development cohort were not eligible for inclusion in the temporal validation cohort, ensuring complete independence between the 2 datasets. The intervals between assessments reflected the original CHARLS survey schedule. Following participant selection, 548 individuals were included in the development cohort and 523 in the temporal validation cohort. The participant selection procedure is illustrated in Figure 1.

### 2.3. Variables and feature selection

Candidate variables included demographic characteristics (age, sex, marital status, educational attainment, and household registration status), clinical history (smoking status, alcohol use, diabetes, chronic lung disease, liver disease, kidney disease, and gastric disease), laboratory biomarkers (lipid profile, renal function indicators, C-reactive protein, hematological parameters, and fasting glucose), and composite indicators (mean right-hand grip strength, total household income, per capita household consumption, average daily sleep duration, cognitive score, and triglyceride-glucose [TyG] index). Cognitive score in CHARLS was assessed using a composite score consisting of mental status and episodic memory (total range, 0–21).^[9]^ Mental status included orientation, calculation, and drawing ability (0–11 points). Episodic memory was assessed using a 10-word recall test (0–10 points).^[10]^ Higher scores indicated better cognitive performance. The TyG index was estimated by measuring fasting triglyceride and fasting plasma glucose levels as follows: TyG index = ln (fasting TG [mg/dL] × fasting plasma glucose [mg/dL]/2). The TyG index is a non-insulin-based surrogate marker of insulin resistance that is straightforward to calculate and combines both abnormal glucose metabolism and lipid metabolism. A higher TyG index value usually means a higher rate of insulin resistance and a greater degree of metabolic dysregulation. As was shown in previous studies, the TyG index has been found to detect insulin resistance and is highly correlated with insulin sensitivity measured by the hyperinsulinemic-euglycemic clamp.^[11]^ Variables with ≥30% missing data were excluded. For the remaining variables, missing values were imputed using a random forest (RF)-based multiple imputation method.

**Figure 2.**
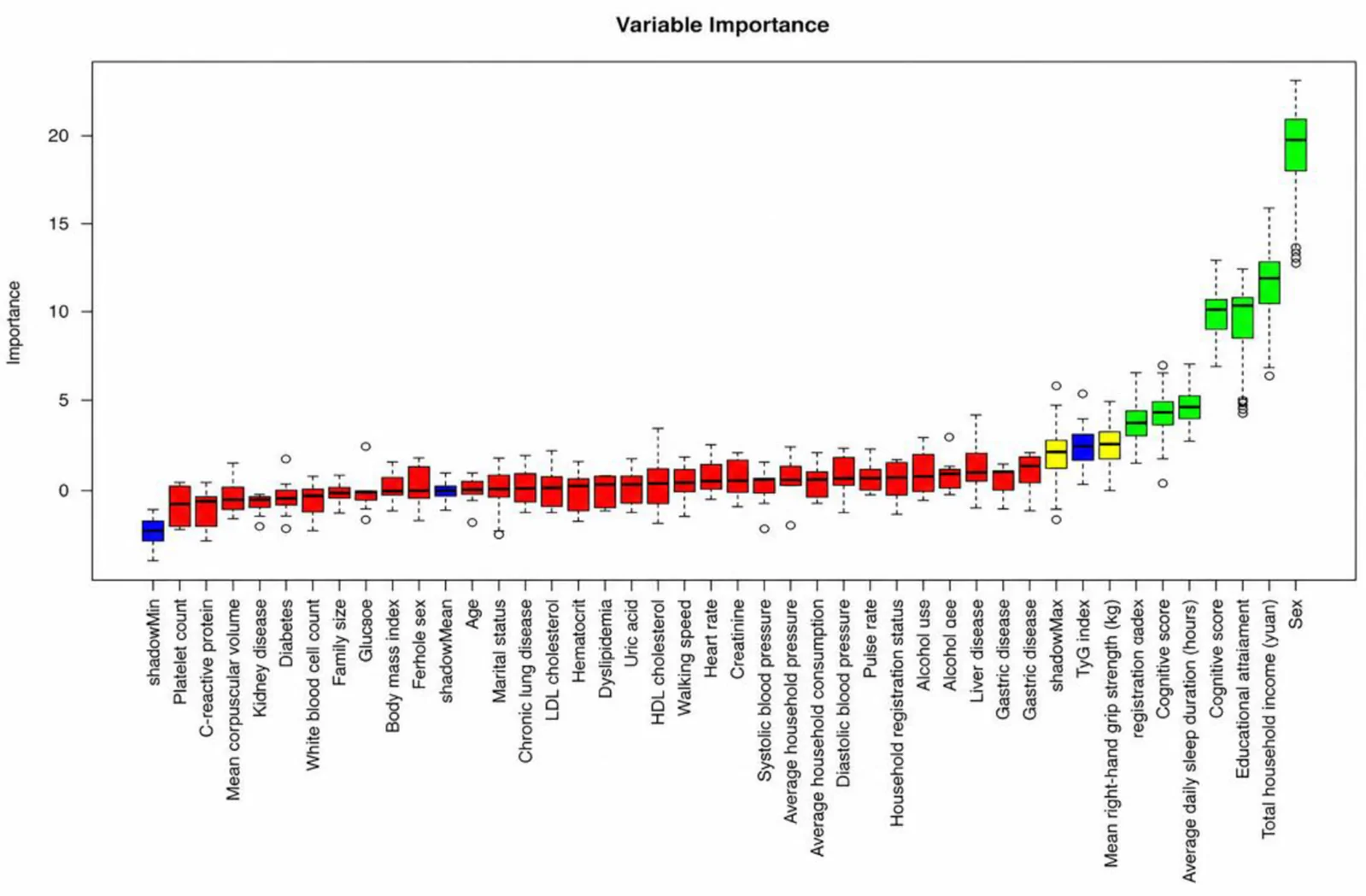
Feature selection using the Boruta algorithm. Green box plots represent confirmed predictors, yellow box plots represent tentative predictors, red box plots represent rejected predictors, and blue box plots represent shadow features. HDL = high-density lipoprotein cholesterol, LDL = low-density lipoprotein cholesterol, TyG = triglyceride-glucose.

The Boruta algorithm was applied to the development cohort for feature selection. Boruta is a RF-based wrapper method that identifies all relevant features by comparing original variables with permuted shadow features. Seven predictors were finally selected: sex, educational attainment, mean right-hand grip strength, TyG index, total household income, average daily sleep duration, and cognitive score. A schematic diagram of feature variable selection is shown in Figure 2. The definition table of categorical variables is shown in Table 1.

**Table 1:** Definitions of categorical variables.

Variable	Coding
Sex	Male = 1; Female = 2
Educational attainment	Junior high school and below = 0; High school and above = 1
Household registration status	Urban = 0; Rural = 1
Marital status	Married and living together = 0; Other marital status = 1
Alcohol use	No = 0; Yes = 1
Smoking status	No = 0; Yes = 1
Disease history	No = 0; Yes = 1

### 2.4. Outcome definition

Depressive symptoms were assessed using the CES-D-10 in CHARLS. The CES-D-10 is a widely used screening tool with good reliability and validity.^[12,13]^ Total scores range from 0 to 30, with higher scores indicating more severe depressive symptoms.^[14]^ A cutoff score of ≥10 was used to define depressive symptoms, consistent with previous epidemiological studies.

### 2.5. Model development and validation

Seven machine learning algorithms were used to develop prediction models, including logistic regression (LR), k-nearest neighbors, support vector machine, RF, extreme gradient boosting, decision tree, and gradient boosting decision tree. Model performance was evaluated using the area under the receiver operating characteristic curve (AUC), as well as accuracy, sensitivity, specificity, and F1 score. Decision curve analysis was used to assess clinical utility. The best-performing model in the development cohort was selected and independently validated in the temporal validation cohort with fixed parameters.

### 2.6. Model interpretation

To improve interpretability, Shapley additive explanations (SHAP) analysis was used to quantify the contribution of each feature to model predictions. SHAP values were calculated based on cooperative game theory. The mean absolute SHAP value was used to assess global feature importance, while individual-level SHAP values were used to explain model predictions across samples.

### 2.7. Statistical analysis

Variables with ≥30% missing values were excluded from the analysis. For variables with <30% missing data, multiple imputation was performed using the RF algorithm. All statistical analyses were performed using R version 4.2.3 (R Foundation for Statistical Computing) and Python version 3.11.4 (Python Software Foundation). A two-sided *P* value < .05 was considered statistically significant. Normally distributed continuous variables were expressed as mean ± standard deviation, and between-group comparisons were conducted using the independent-samples *t* test. Non-normally distributed variables were presented as median (interquartile range) and compared using the Mann–Whitney *U* test. Categorical variables were expressed as counts (percentages) and analyzed using the Pearson chi-square test or Fisher’s exact test, as appropriate.

## 3. Results

### 3.1. Baseline characteristics

Following the screening process, a total of 1071 participants were included in this study, comprising 548 participants in the development cohort and 523 in the temporal validation cohort. In the development cohort, 131 participants (23.9%) developed depressive symptoms during follow-up. The median age of participants in the development cohort was 66 years. Regarding sex distribution, there were 330 males (60.2%) and 218 females (39.8%). Additional baseline characteristics, including family situation, vital signs, and laboratory indicators, are provided in Table 2. Several baseline characteristics differed significantly between the 2 groups. Participants who developed long-term depressive symptoms were more likely to be female, exhibit lower mean right-hand grip strength, have a lower prevalence of alcohol use, and report lower total household income; educational attainment did not differ significantly between groups (*P* = .054). Regarding biochemical indicators, participants who developed long-term depressive symptoms had a higher composite TyG index. Finally, we found that participants who developed depressive symptoms had shorter average daily sleep duration and lower cognitive scores.

**Table 2:** Baseline characteristics of the development cohort.

Variable	Category	Overall (n = 548)	Without depressive symptoms (n = 417)	Developed depressive symptoms (n = 131)	Statistic	*P* value
Sex, n (%)	Male	330 (60.219)	296 (70.983)	34 (25.954)	84.373	<.001
	Female	218 (39.781)	121 (29.017)	97 (74.046)		
Marital status, n (%)	Married and living together	445 (81.204)	339 (81.295)	106 (80.916)	0.009	.923
	Other marital status	103 (18.796)	78 (18.705)	25 (19.084)		
Household registration status, n (%)	Urban	166 (30.292)	139 (33.333)	27 (20.611)	7.641	.006
	Rural	382 (69.708)	278 (66.667)	104 (79.389)		
Educational attainment, n (%)	Junior high school and below	475 (86.679)	368 (88.249)	107 (81.679)	3.727	.054
	High school and above	73 (13.321)	49 (11.751)	24 (18.321)		
Alcohol use, n (%)	No	355 (64.781)	253 (60.671)	102 (77.863)	12.913	<.001
	Yes	193 (35.219)	164 (39.329)	29 (22.137)		
Smoking status, n (%)	No	365 (66.606)	273 (65.468)	92 (70.229)	1.016	.313
	Yes	183 (33.394)	144 (34.532)	39 (29.771)		
Diabetes, n (%)	No	442 (80.657)	338 (81.055)	104 (79.389)	0.177	.674
	Yes	106 (19.343)	79 (18.945)	27 (20.611)		
Gastric disease, n (%)	No	472 (86.131)	363 (87.050)	109 (83.206)	1.233	.267
	Yes	76 (13.869)	54 (12.950)	22 (16.794)		
Chronic lung disease, n (%)	No	482 (87.956)	372 (89.209)	110 (83.969)	2.583	.108
	Yes	66 (12.044)	45 (10.791)	21 (16.031)		
Dyslipidemia, n (%)	No	463 (84.489)	350 (83.933)	113 (86.260)	0.412	.521
	Yes	85 (15.511)	67 (16.067)	18 (13.740)		
Arthritis, n (%)	No	389 (70.985)	304 (72.902)	85 (64.885)	3.110	.078
	Yes	159 (29.015)	113 (27.098)	46 (35.115)		
Kidney disease, n (%)	No	527 (96.168)	402 (96.403)	125 (95.420)	0.261	.609
	Yes	21 (3.832)	15 (3.597)	6 (4.580)		
Age (yr)		66.000 (63.000, 71.000)	66.000 (63.000, 71.000)	67.000 (63.000, 71.000)	−0.677	.498
Family size (persons)		2.000 (2.000, 5.000)	2.000 (2.000, 5.000)	2.000 (2.000, 5.000)	0.527	.579
Mean systolic blood pressure (mm Hg)		147.330 (136.670, 162.330)	146.670 (136.000, 160.670)	150.000 (137.330, 166.330)	−1.876	.061
Mean diastolic blood pressure (mm Hg)		80.000 (71.670, 87.670)	79.670 (71.330, 87.670)	80.330 (73.330, 87.330)	−1.108	.268
Pulse rate (beats/min)		70.670 (63.000, 77.000)	70.330 (62.330, 76.670)	72.000 (64.330, 79.000)	−1.938	.053
Mean right-hand grip strength (kg)		30.100 (23.500, 37.750)	31.950 (24.350, 39.000)	26.250 (20.000, 31.750)	4.714	<.001
Body mass index (kg/m^2^)		23.800 (21.780, 26.050)	23.830 (21.780, 26.120)	23.600 (21.690, 25.800)	0.410	.682
Per capita household consumption (yuan)		5170.000 (2956.000, 8680.000)	5398.000 (2970.000, 9076.000)	4620.000 (2813.330, 7760.000)	1.283	.200
Total household income (yuan)		18,760.000 (3840.000, 35,087.220)	22,260.000 (5970.140, 41,100.000)	6585.000 (1778.000, 21,600.000)	6.162	<.001
TyG index		8.676 (8.460, 8.960)	8.673 (8.430, 8.940)	8.710 (8.654, 9.060)	−3.051	.002
Cognitive score (points)		12.000 (9.500, 14.000)	12.150 (10.140, 14.500)	10.500 (7.500, 12.000)	6.935	<.001
Platelet count (×10^9^/L)		214.867 (177.000, 239.000)	215.349 (178.000, 241.000)	211.000 (177.000, 235.000)	0.943	.346
Mean corpuscular volume (fL)		90.585 (88.228, 94.300)	90.642 (88.200, 94.300)	90.430 (88.254, 94.600)	0.115	.908
White blood cell count (×10^9^/L)		6.139 (5.300, 6.800)	6.160 (5.310, 6.800)	6.100 (5.200, 7.000)	0.299	.765
Hematocrit (%)		40.000 (36.970, 44.400)	40.000 (37.000, 44.700)	39.500 (36.711, 43.800)	1.178	.239
C-reactive protein (mg/L)		1.750 (0.950, 2.700)	1.760 (0.960, 2.759)	1.670 (0.880, 2.519)	0.473	.636
Uric acid (mg/dL)		4.633 (4.070, 5.200)	4.652 (4.090, 5.220)	4.585 (3.970, 4.953)	1.286	.199
Creatinine (mg/dL)		0.816 (0.730, 0.900)	0.835 (0.745, 0.920)	0.793 (0.690, 0.866)	3.208	.001
Blood urea nitrogen (mg/dL)		16.265 (14.230, 18.090)	16.330 (14.590, 18.290)	16.080 (13.050, 17.110)	2.225	.026
Low-density lipoprotein cholesterol (mg/dL)		118.802 (106.320, 133.380)	118.822 (104.380, 133.380)	118.690 (112.110, 136.470)	−0.594	.553
High-density lipoprotein cholesterol (mg/dL)		52.926 (43.300, 56.579)	52.960 (42.910, 56.745)	52.190 (43.690, 56.440)	0.309	.758
Average daily sleep duration (h)		7.000 (6.000, 8.000)	7.000 (6.000, 8.000)	6.000 (5.000, 7.000)	3.788	<.001

TyG = triglyceride-glucose.

### 3.2. Performance comparison of 7 machine learning algorithms

We conducted internal validation through 10-fold cross-validation on the development cohort and assessed the performance of 7 machine learning models in predicting long-term depressive symptoms in older adults with CVD or hypertension.

**Figure 3.**
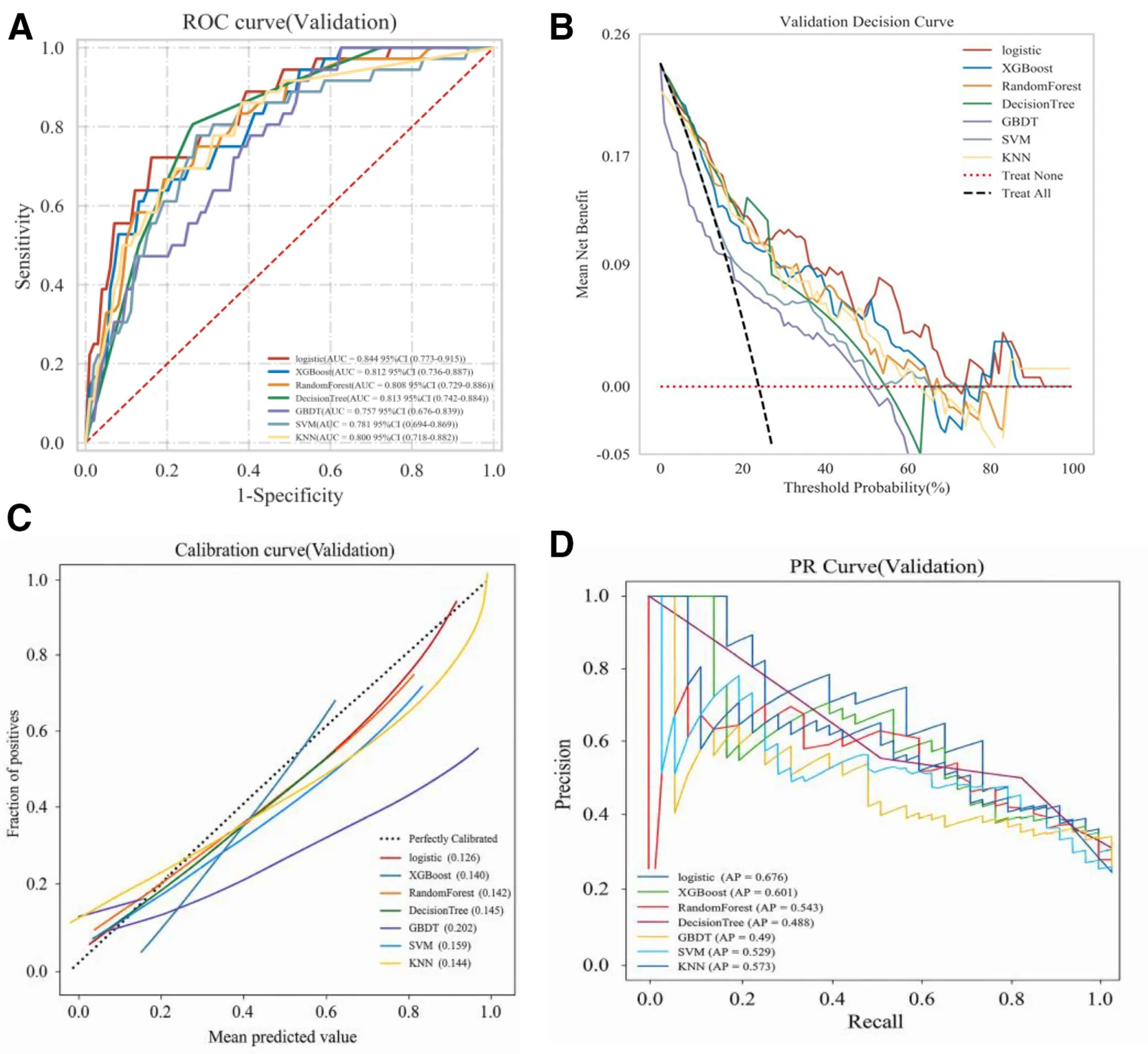
Predictive performance of 7 machine learning models: (A) receiver operating characteristic curves; (B) decision curves; (C) calibration curves; and (D) precision-recall curves. In the software-generated panel titles, “Validation” refers to the development cohort. AP = average precision, AUC = area under the receiver operating characteristic curve, GBDT = gradient boosting decision tree, KNN = k-nearest neighbors, PR = precision-recall, ROC = receiver operating characteristic, SVM = support vector machine, XGBoost = extreme gradient boosting.

In the analysis of the receiver operating characteristic (ROC) curve (Fig. 3A), the LR model demonstrated outstanding predictive performance, achieving an AUC of 0.844, signifying high accuracy. In comparison, the AUC values for the other models were as follows: extreme gradient boosting, 0.812; RF, 0.808; and decision tree, 0.813.

Decision curve analysis (Fig. 3B) assessed the clinical utility of the models and revealed that the logistic model provided the highest net benefit across most threshold ranges, particularly in the intermediate range, highlighting its superiority in predicting long-term outcomes.

Calibration analysis demonstrated that the LR model showed the best agreement between predicted and observed probabilities and achieved the lowest Brier score (0.126) among all candidate models (Fig. 3C).

On the precision-recall (PR) curve (Fig. 3D), the LR model had the highest average precision (0.676), indicating a better PR trade-off than the other candidates.

Across ROC, calibration, decision curve, and PR analyses, the LR model consistently outperformed the other candidate models and was therefore selected as the optimal model for subsequent interpretation and validation. More detailed performance metrics for each model, including sensitivity, specificity, AUC, accuracy, and F1 score, are presented in Table 2. Detailed performance metrics for each model are presented in Table 3.

**Table 3:** Comparison of detailed performance metrics of each model.

Model	AUC (95% CI)	Cutoff	Accuracy	Sensitivity	Specificity	PPV	NPV	F1 score	Kappa
Logistic regression	0.844 (0.773–0.915)	0.207	0.715	0.778	0.696	0.444	0.909	0.566	0.376
XGBoost	0.812 (0.736–0.887)	0.242	0.722	0.694	0.730	0.446	0.884	0.543	0.357
Random forest	0.808 (0.729–0.886)	0.600	0.795	0.333	0.939	0.632	0.818	0.436	0.325
Decision tree	0.813 (0.742–0.884)	0.268	0.755	0.806	0.739	0.492	0.924	0.611	0.447
GBDT	0.757 (0.676–0.839)	0.143	0.689	0.556	0.730	0.392	0.840	0.460	0.250
SVM	0.781 (0.694–0.869)	0.366	0.735	0.694	0.748	0.463	0.887	0.556	0.377
KNN	0.800 (0.718–0.882)	1.000	0.775	0.056	1.000	1.000	0.772	0.105	0.082

AUC values are reported with 95% confidence intervals; the remaining performance metrics are presented as point estimates.

AUC = area under the receiver operating characteristic curve, CI = confidence interval, GBDT = gradient boosting decision tree, KNN = k-nearest neighbors, NPV = negative predictive value, PPV = positive predictive value, SVM = support vector machine, XGBoost = extreme gradient boosting.

DeLong testing showed that the LR model had a significantly higher AUC than the gradient boosting decision tree model (*P* = .022; Table 4). For the other models, the differences compared with LR were not significant (all *P* > .05).

**Table 4:** *P* values from pairwise DeLong tests for predictive performance of each model.

Model category	Logistic regression	XGBoost	Random forest	Decision tree	GBDT	SVM	KNN
Logistic regression	NA	0.165	0.099	0.320	0.022	0.053	0.117
XGBoost	0.165	NA	0.860	0.961	0.195	0.347	0.732
Random forest	0.099	0.860	NA	0.852	0.231	0.354	0.786
Decision tree	0.320	0.961	0.852	NA	0.212	0.231	0.730
GBDT	0.022	0.195	0.231	0.212	NA	0.624	0.316
SVM	0.053	0.347	0.354	0.231	0.624	NA	0.592
KNN	0.117	0.732	0.786	0.730	0.316	0.592	NA

GBDT = gradient boosting decision tree, KNN = k-nearest neighbors, SVM = support vector machine, XGBoost = extreme gradient boosting.

**Figure 4.**
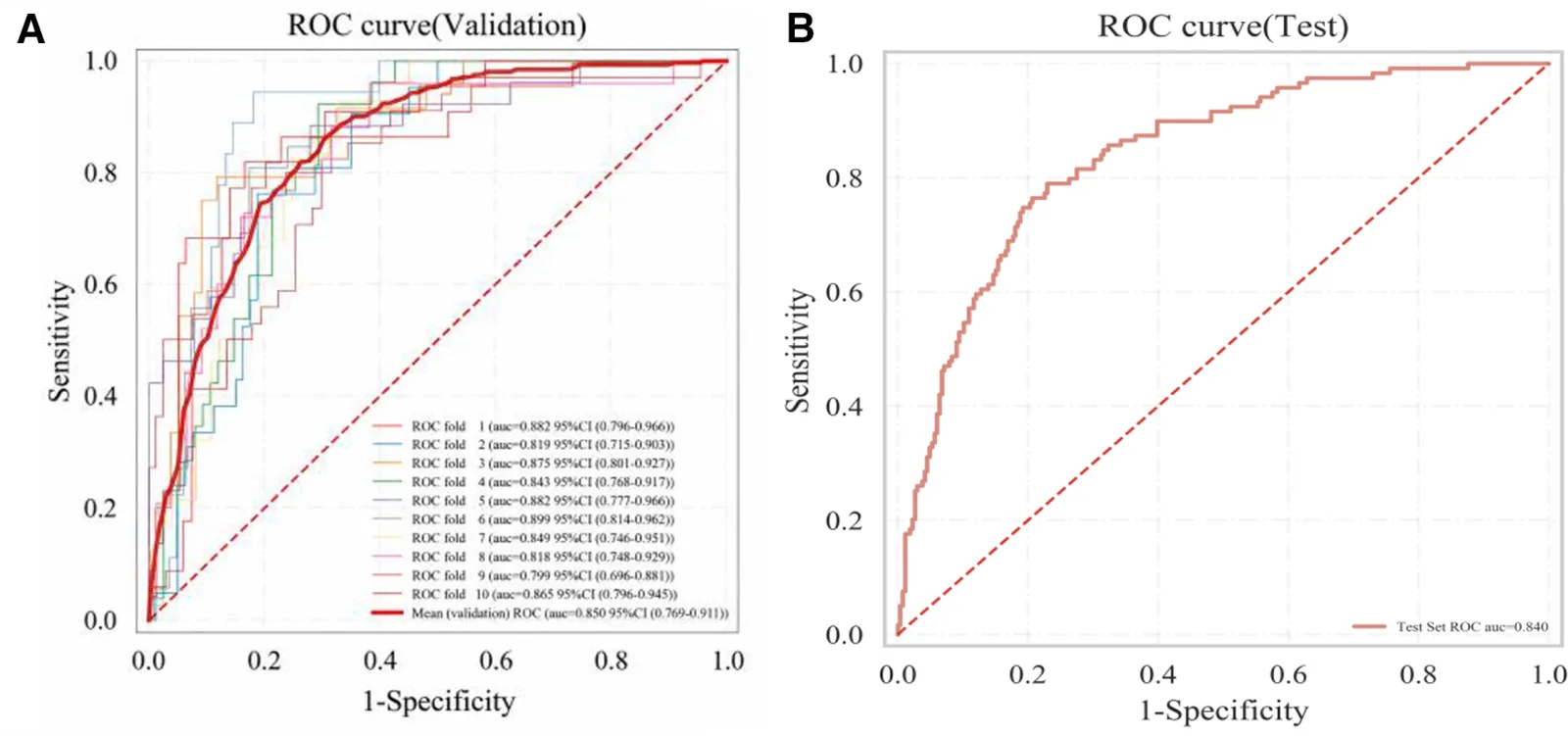
Internal and temporal validation results of the logistic regression model: (A) 10-fold cross-validation in the development cohort; and (B) temporal validation in the 2015 to 2020 cohort. AUC = area under the receiver operating characteristic curve, CI = confidence interval, ROC = receiver operating characteristic.

To further evaluate the stability of the LR model, we plotted the 10-fold cross-validation ROC curve within the development cohort (Fig. 4A), which demonstrated excellent discriminative ability. The LR model also performed well on the 2015 to 2020 temporal validation cohort, achieving an AUC of 0.840 (Fig. 4B).

**Figure 5.**
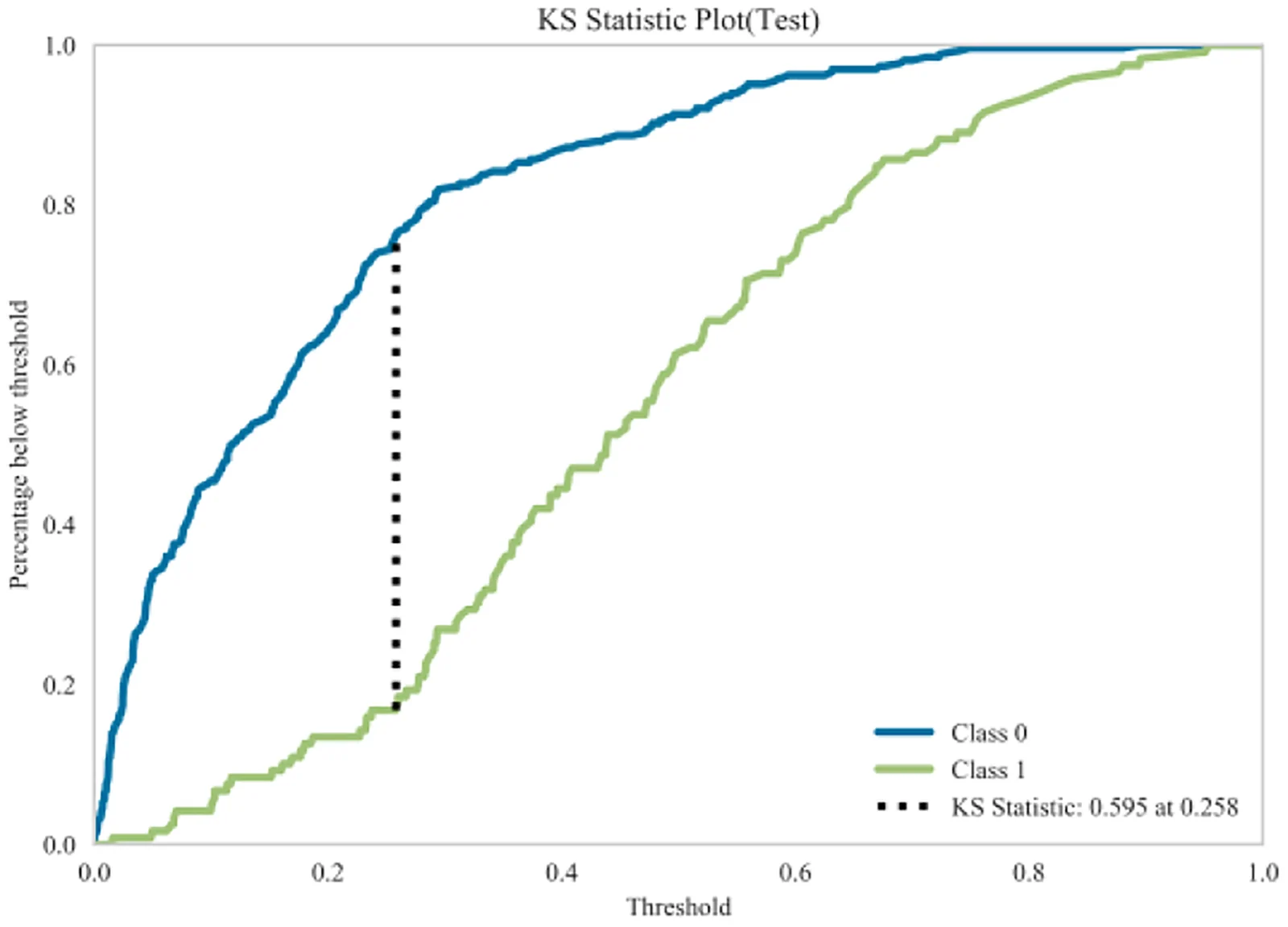
K–S statistic plot for the temporal validation cohort. K–S = Kolmogorov–Smirnov.

**Figure 6.**
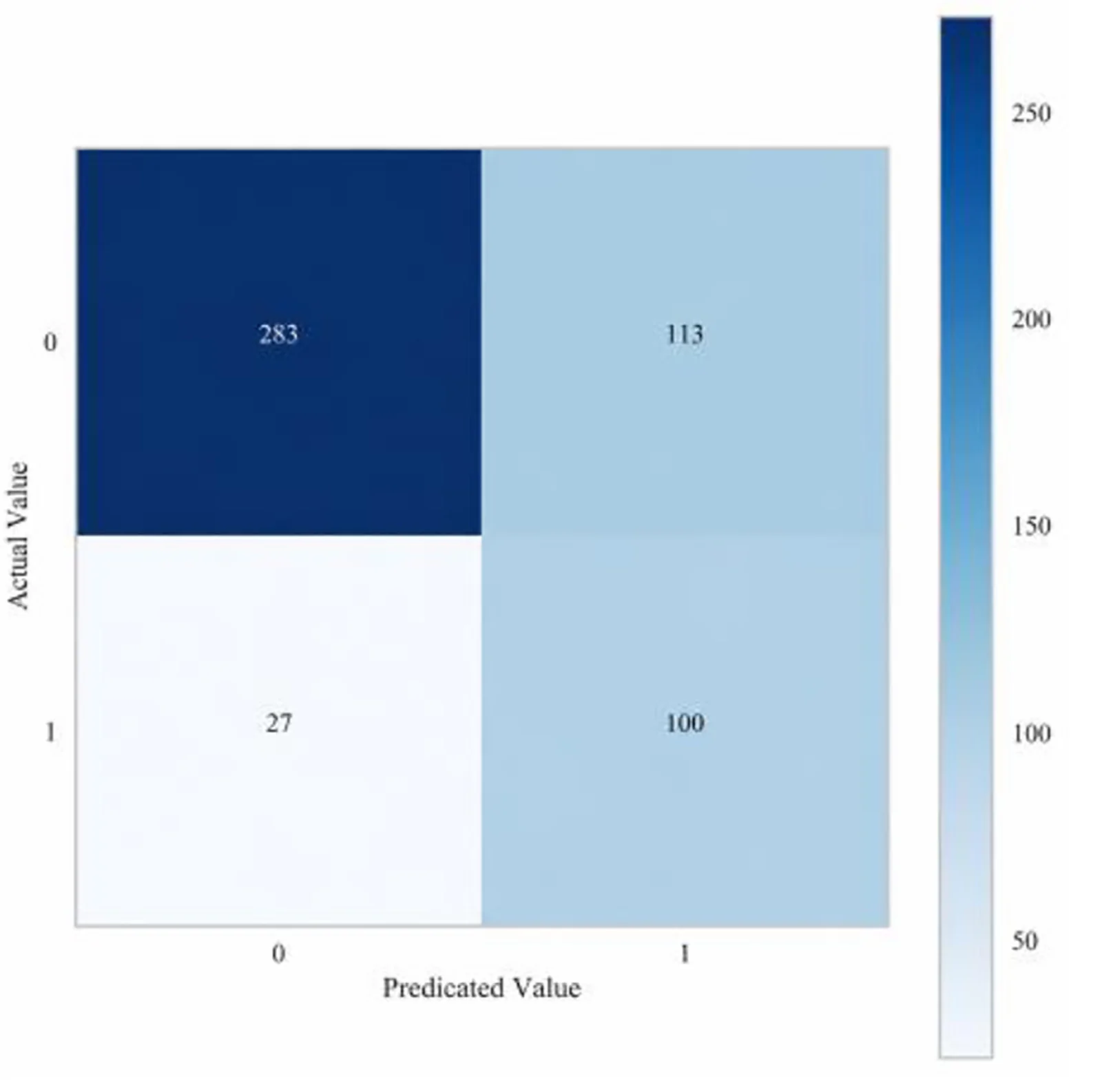
Confusion matrix for the temporal validation cohort.

We also provided the Kolmogorov–Smirnov statistic plot for the temporal validation cohort to assess the model’s discriminative power; the Kolmogorov–Smirnov statistic was 0.595, falling between good and excellent, indicating strong discriminative ability (Fig. 5). Finally, to visually present its classification performance, the confusion matrix of the LR model on the temporal validation cohort (Fig. 6) further demonstrates the model’s superiority.

### 3.3. SHAP

To further interpret the contribution of individual features in the optimal predictive model (LR) for depressive symptoms, SHAP was applied for model interpretability analysis. This approach enables a more clinically meaningful interpretation of model outputs by quantifying both the direction and magnitude of each feature’s contribution at global and individual levels.

#### 3.3.1. SHAP summary plot

**Figure 7.**
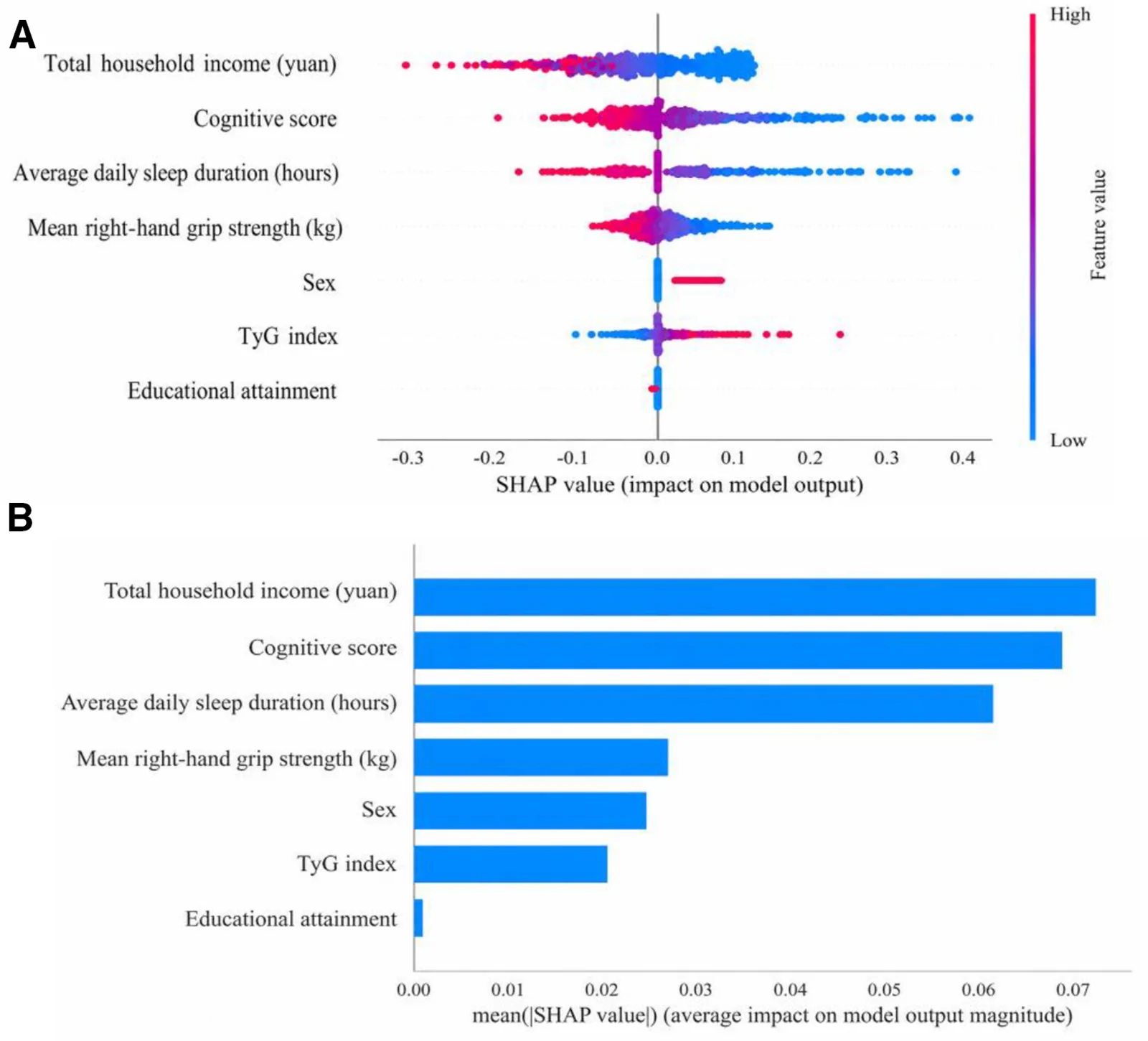
SHAP (Shapley additive explanations) analysis of the model. (A) SHAP summary plot visualizing the distribution of SHAP values for each feature. Each dot represents an individual data point, with the x-axis showing the SHAP value (feature’s impact on the model output) and the color representing the feature value (red for higher values, blue for lower values). (B) Bar plot showing the mean absolute SHAP values for all 7 predictors ranked by their contribution to predicting depressive symptoms in older adults with CVD or hypertension. CVD = cardiovascular disease, TyG = triglyceride-glucose.

Figure 7A presents the overall distribution of feature effects on model output. Each point represents a single sample, with color indicating the feature value (red for higher values and blue for lower values). The x-axis corresponds to SHAP values. Positive SHAP values increase the model-predicted risk of depressive symptoms, whereas negative values decrease the model-predicted risk. The results show that higher levels of total household income, cognitive score, mean right-hand grip strength, and average daily sleep duration were predominantly associated with negative SHAP values, suggesting a lower predicted probability of depressive symptoms. In contrast, higher TyG index values were mainly associated with positive SHAP values, indicating an increased predicted risk of depressive symptoms. Being female is associated with a higher predicted risk. Educational attainment showed a relatively limited impact on model output, with most SHAP values distributed close to 0. These findings are generally consistent with established clinical evidence. For example, lower mean right-hand grip strength and lower socioeconomic status are often linked to frailty and poorer mental health outcomes in older adults, while elevated TyG index may reflect insulin resistance and systemic metabolic dysregulation, both of which have been implicated in depressive symptom development.

#### 3.3.2. SHAP feature-importance ranking

Figure 7B shows the SHAP feature-importance ranking. Among the 7 predictors, total household income had the largest global contribution, with a mean absolute SHAP value of approximately 0.073, followed by cognitive score (approximately 0.069) and average daily sleep duration (approximately 0.061). Educational attainment had a smaller overall contribution to model prediction than mean right-hand grip strength, sex, and the TyG index. From a clinical and public health perspective, total household income, cognitive function, and average daily sleep duration can be considered indicators of socioeconomic resources, neurocognitive status, and daily health-related behavior, respectively, which could potentially impact the long-term risk of depressive symptoms in older adults with CVD or hypertension.

#### 3.3.3. SHAP dependence plots

**Figure 8.**
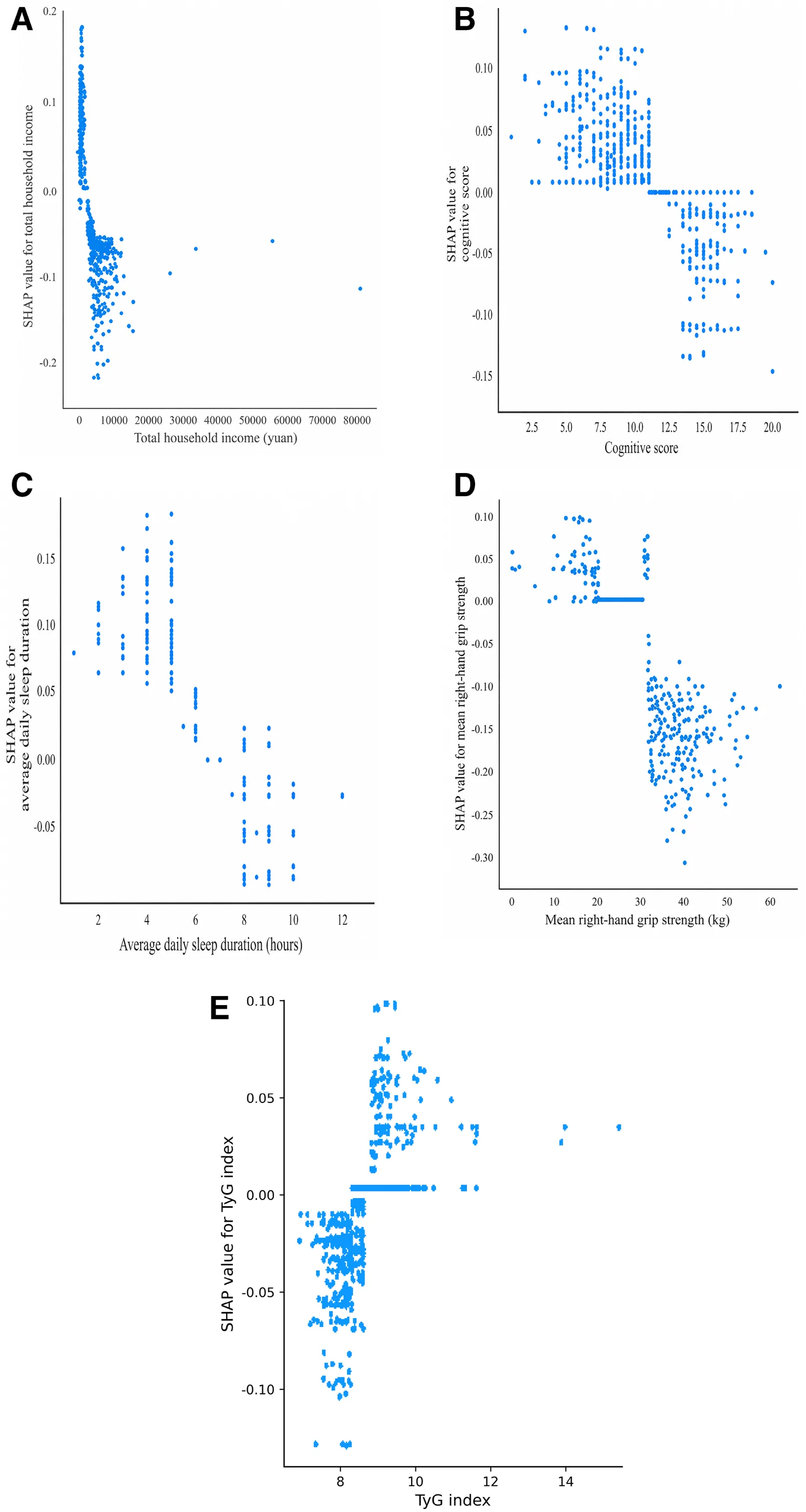
SHAP dependence plots: (A) total household income; (B) cognitive score; (C) average daily sleep duration; (D) mean right-hand grip strength; and (E) TyG index. SHAP = Shapley additive explanations, TyG = triglyceride-glucose.

As shown in Figure 8, apparent nonlinear patterns were observed on the SHAP scale between several features (mean right-hand grip strength, average daily sleep duration, total household income, cognitive score, and TyG index) and model output. Higher levels of mean right-hand grip strength, average daily sleep duration, and total household income were associated with decreasing SHAP values. Cognitive score showed a threshold-like pattern, where lower values were associated with a positive contribution to risk prediction, whereas higher values showed a negative contribution. For the TyG index, lower values were mainly associated with negative SHAP values, while increasing levels gradually shifted toward a positive contribution to the predicted risk. It should be noted that these patterns reflect associative, rather than causal, relationships. These dependence plots do not by themselves establish interaction effects; SHAP interaction values or interaction-colored dependence plots would be required to support such an interpretation.

#### 3.3.4. Development of a dynamic nomogram based on the logistic regression model

**Figure 9.**
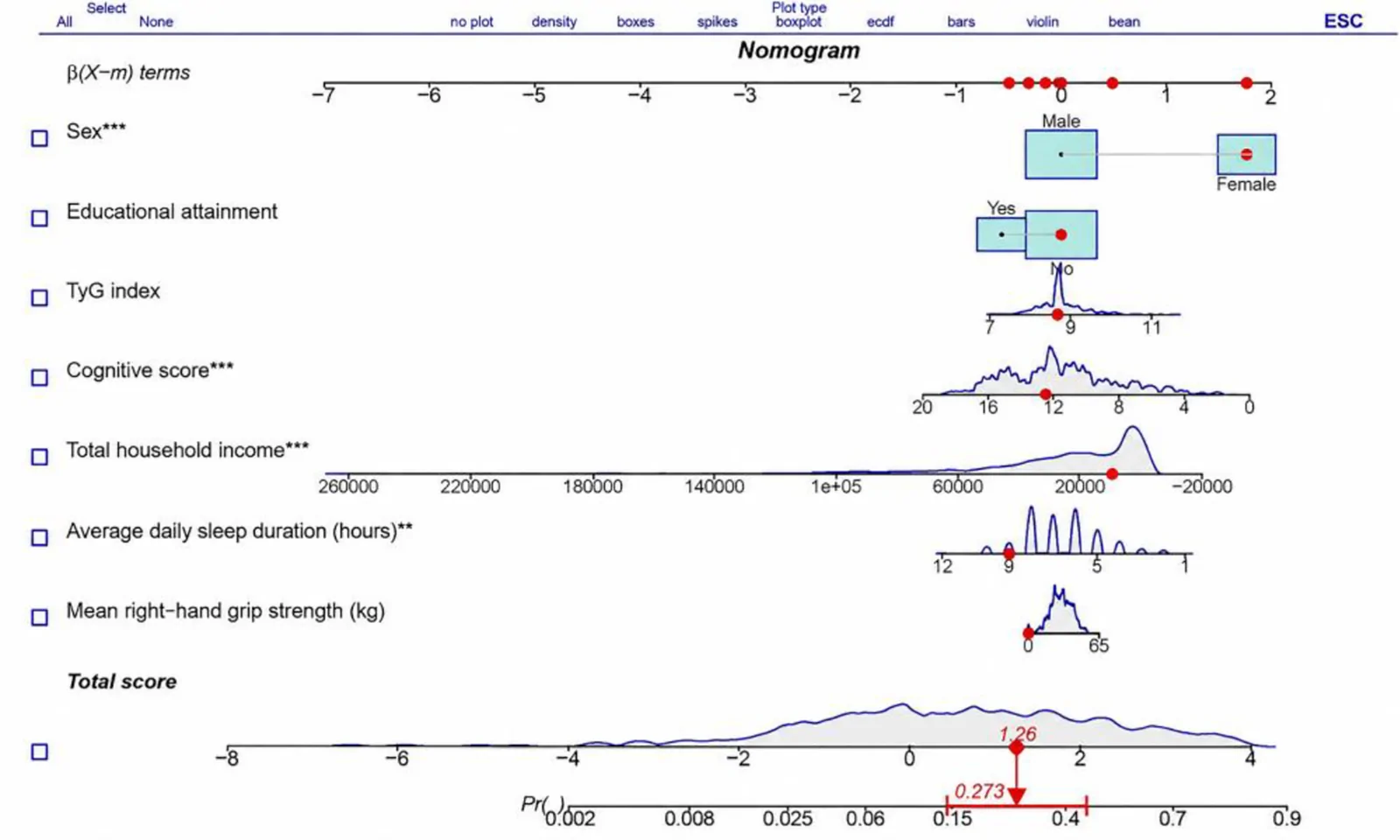
The dynamic nomogram based on the logistic regression model. The nomogram used sex, educational attainment, TyG index, cognitive score, total household income, average daily sleep duration, and mean right-hand grip strength to give a person-specific prediction of the probability of developing depressive symptoms. The red point represents the predictor value of an example person, and the blue lines or boxes represent the distribution of the variable being studied in the sample population. Predictor contributions were summed to obtain the total score; in the given scenario, a score of 1.26 was associated with a predicted probability of 27.3%. TyG = triglyceride-glucose.

After comparing the predictive performance of the candidate machine learning models, LR showed the best overall performance and was selected as the final model. A dynamic nomogram was created thereafter using sex, educational attainment, TyG index, cognitive score, total household income, average daily sleep duration, and mean right-hand grip strength to enable the visualization and individualized estimation of the risk of depressive symptoms. Each predictor contributes according to its observed value; these contributions are summed to generate a total score, which is then converted into a predicted probability. As shown in Figure 9, the representative individual scored 1.26, which indicates a predicted outcome probability of 27.3%. Such results suggest that the dynamic nomogram can be used as an intuitive device in individualized risk stratification and clinical decision-making.

## 4. Discussion

Older adults with CVD or hypertension are approximately 2 to 3 times more likely to experience depressive symptoms compared with their same-aged peers,^[15]^ which greatly impacts their prognosis and quality of life. Considering the significant effect of CVD on global mortality and morbidity,^[16]^ it is essential to create a predictive model suitable for clinical or community hospital environments. This will facilitate early identification and effective intervention, ultimately lowering the risk of depressive symptoms in this population.

Currently, research on predictive models for long-term risk of depressive symptoms in older adults with CVD or hypertension remains limited.^[17]^ Zhang et al created an online screening tool utilizing machine learning to identify depressive symptoms in patients with cardiovascular and cerebrovascular disease (CCVD),^[18]^ based on cross-sectional data from the CHARLS 2020 survey. Zhang et al^[19]^ established a machine learning model to predict depression risk in patients with cardiometabolic diseases. However, most of these studies used single-year cross-sectional data, lacking a systematic consideration of the temporal dimension, and did not fully utilize the multi-wave longitudinal follow-up data of CHARLS to capture the temporal relationship between disease exposure and long-term depressive outcomes. In contrast, this study focused on a broader population of older adults with CVD or hypertension and, to our knowledge, is among the first studies to utilize longitudinal CHARLS data spanning 2011 to 2020. We constructed the prediction model using 2011 to 2015 data (development cohort) and independently validated its performance using 2015 to 2020 data (temporal validation cohort), thereby achieving rigorous temporal validation. This design not only addresses the lack of temporal validation in previous models but also provides evidence supporting the temporal robustness of the model across different follow-up periods, suggesting satisfactory generalizability over time. By establishing and validating the model in 2 nonoverlapping time windows, this study significantly reduced the risk of overfitting, enhanced the credibility of the prediction model, and provided a more practically valuable predictive tool for the early identification and clinical intervention of long-term risk of depressive symptoms in older adults with CVD or hypertension.

For the clinical prediction model, we used the Boruta algorithm to select 7 risk factors for inclusion. We incorporated many easily obtainable clinical indicators that have been widely reported to be associated with depressive symptoms. Factors such as female sex, lower educational attainment, lower total household income, and shorter average daily sleep duration were substantially linked to depressive symptoms.^[20–22]^ We also included several novel composite indicators (TyG index, cognitive score, and mean right-hand grip strength) that have been reported to be associated with depressive symptoms^[23,24]^ but have rarely been incorporated into predictive models; their inclusion improved the model’s predictive performance.

Studies have shown that women are more psychologically sensitive to negative life events (especially those occurring within the family) and experience higher levels of social isolation.^[25]^ Conversely, individuals with higher levels of education typically have greater cognitive reserve, allowing them to better manage cognitive changes that occur after CVD or hypertension events.^[22,26]^ This enhanced coping ability helps them maintain a positive view of their health, which can reduce the risk of depression. On the other hand, individuals with lower levels of education typically exhibit poorer mental health literacy, less ability to recognize symptoms, and a reduced willingness to seek assistance, potentially resulting in prolonged periods of unrecognized and untreated depression. The TyG index has recently become a subject of widespread interest as an easy-to-use and practical surrogate indicator of insulin resistance that combines abnormalities in glucose and lipid metabolism. Insulin resistance in elderly people with CVD or hypertension can be an important factor in the onset of depressive symptoms due to vascular dysfunction, decreased brain metabolism, and disturbances in insulin signaling pathways. As demonstrated by earlier research, a higher TyG index is linked to depressive symptoms among hypertensive individuals, whereas insulin resistance has also been found to be an independent risk factor for depression following stroke.^[27,28]^ Hence, the predictive contribution of the TyG index in our model might indicate common metabolic pathophysiologic processes that underlie CVD and depressive symptoms.

The composite indicators can largely be obtained through questionnaires, face-to-face computer-assisted interviews, or home testing,^[29]^ greatly enhancing the practical utility of the prediction model. In this study, we included cognitive score as a composite indicator, a unique assessment tool in the CHARLS database that endows the model with stronger early-warning capability. The distinctive value of cognitive score lies in its “upstream warning” characteristic – subtle changes in cognitive function may serve as a “sentinel signal” for the risk of depressive symptoms before individuals exhibit obvious emotional symptoms. LR performed better than more complex machine learning algorithms, suggesting that the relationships between predictors and depressive symptoms may be relatively stable and interpretable in this population. Ji et al^[30]^ used 4 waves of CHARLS data from 2013 to 2020 and applied dynamic time warping, cross-lagged panel models, and network analysis to strongly support the “cognition-first” hypothesis. They found that declines in cognitive domains such as temporal orientation, calculation ability, and immediate recall were significant upstream predictors of subsequent depressive symptoms. This finding methodologically elucidates the deeper logic behind why cognitive score enhances prediction of depressive symptoms: cognitive changes are not only concurrent with depressive symptoms but may also precede clinical symptom manifestation along the timeline.

SHAP analysis improved model interpretability by quantifying each predictor’s contribution to the output.^[31]^ Total household income was the most influential feature, followed by cognitive score and average daily sleep duration. These results emphasize the contributions of socioeconomic status, cognitive function, sleep, and physical function to model predictions, supporting the model’s clinical plausibility.

The 7 predictors in the nomogram might be applied by a clinician or community health worker to predict the likelihood of long-term depressive symptoms in older adults during actual practice. People who have a projected probability of 0.207 or above may be referred for more intensive follow-up and repeated CES-D-10 tests. A person who has a score of 10 or higher on the CES-D-10 or any other clinical symptoms needs to be further examined psychologically and, where necessary, referred to a specialist. Nonetheless, the 0.207 cutoff point is an interim screening threshold, not a diagnostic requirement. The nomogram ought to be considered a decision-support tool, and it should be validated prospectively and recalibrated locally prior to its routine application.

Despite the favorable performance of the prediction model, several limitations should be acknowledged. First, both CVD and hypertension were determined by self-reported physician diagnosis and medication-use information, as reported in the Methods, and not by medical records or physician-adjudicated clinical diagnosis. Despite the fact that this method is often applied in large population-based studies, it might have introduced recall bias and disease misclassification.^[32]^ In addition, due to the standardized format of the CHARLS questionnaire, it was impossible to identify certain subtypes of CVD, including peripheral vascular disease and valvular heart disease, and hence they were excluded from the composite definition.^[33]^ Second, the CES-D-10 screening scale was used to determine the presence of depressive symptoms instead of a structured clinical diagnostic interview, which could have resulted in recall bias or misclassification of outcomes.^[34,35]^ Third, due to the old age of the sample population and the long-term follow-ups, there were no guarantees against survivorship bias and selective attrition. Individuals with extreme levels of CVD, severe frailty, or depressive patterns might have had higher chances of dying or dropping out prior to follow-up, leaving behind a healthier analytical cohort and a lower representation of the most vulnerable members of the study population. As a result, the model may underestimate the severity of depressive symptoms among such high-risk people, and its discrimination and calibration may be poorer compared with what is seen in the entire study population. Although the model showed consistent performance in 2 nonoverlapping temporal cohorts, its forecasting accuracy in persons with severe CVD, extreme frailty, or an elevated risk of early death should be interpreted with caution and needs further testing.

However, none of these constraints reduce the possible clinical and population health benefits that can be provided by the current results. The best model showed great discrimination, calibration, and clinical net benefit in both the development and temporal validation cohorts, which means good predictive stability and temporal transportability between different survey periods. Moreover, a nomogram was derived to enable individualized and visually understandable risk estimation with the help of a small set of routinely available predictors. Because it is associated with low implementation load, predictor availability, and positive interpretability, the model could function as a useful addition to the psychological screening approach currently in place instead of replacing the conventional clinical assessment. Taken together, this model would be a helpful instrument in determining which older people with CVD or hypertension are more likely to develop depressive symptoms, so that risk stratification, targeted screening, and timely preventive measures can be implemented.

## 5. Conclusion

In this study, multiple machine learning models were developed and compared to predict the long-term risk of depressive symptoms among older adults with CVD or hypertension. Following systematic evaluation, the LR model demonstrated the best predictive performance. Sex, educational attainment, mean right-hand grip strength, the TyG index, total household income, average daily sleep duration, and cognitive score were identified as important predictors of long-term depressive symptoms. Based on the final model, we have also developed a dynamic nomogram that allows the estimation of an individual’s future risk of depressive symptoms in an intuitive manner. The resulting instrument can help physicians and community health workers with risk stratification, focused screening, and the introduction of personalized preventive measures.

## Acknowledgments

This work is supported by the Extreme Smart Analysis platform (https://www.xsmartanalysis.com/).

## Author contributions

**Conceptualization:** Yang Zhao.

**Data curation:** Yang Zhao, Siji Chen, Chang Pang, Yao Tang, Si Long, Hua Zheng, Muhua Zeng, Shuwu Lin.

**Formal analysis:** Yang Zhao.

**Funding acquisition:** Shuwu Lin.

**Investigation:** Chang Pang, Yao Tang, Si Long, Shuwu Lin.

**Methodology:** Yang Zhao, Siji Chen, Hua Zheng, Muhua Zeng, Shuwu Lin.

**Project administration:** Yang Zhao.

**Supervision:** Shuwu Lin.

**Writing – original draft:** Yang Zhao, Siji Chen, Shuwu Lin.

**Writing – review & editing:** Yang Zhao, Shuwu Lin.
